# Flow Pattern Study and Pressure Drop Prediction of Two-Phase Boiling Process in Different Surface Wettability Microchannel

**DOI:** 10.3390/mi14050958

**Published:** 2023-04-28

**Authors:** Yuqi Zhang, Haoxian Wu, Ling Zhang, Yunbo Yang, Xiangdong Niu, Zerong Zeng, Bifen Shu

**Affiliations:** 1 School of Physics, Sun Yat-sen University, Guangzhou 510006, China; zhangyq69@mail2.sysu.edu.cn (Y.Z.);; 2 Shuifa Singyes Energy (Zhuhai) Co., Ltd., Zhuhai 519000, China; 3 Zhuhai CABEE Singyes Green Building Design & Research Institute Co., Ltd., Zhuhai 519000, China

**Keywords:** pressure drop, hydrophilic microchannel, flow order degree, two-phase flow

## Abstract

An experimental study of two-phase flow pressure drop using R-134a is conducted on three types of different surface wettability microchannels with superhydrophilic (contact angle of 0°), hydrophilic (contact angle of 43°) and common (contact angle of 70°, unmodified) surfaces, all with a hydraulic diameter of 0.805 mm. Experiments were conducted using a mass flux of 713–1629 kg/m^2^s and a heat flux of 7.0–35.1 kW/m^2^. Firstly, the bubble behavior during the two-phase boiling process in the superhydrophilic and common surface microchannel is studied. Through a large number of flow pattern diagrams under different working conditions, it is found that the bubble behavior shows different degrees of order in microchannels with different surface wettability. The experimental results show that the hydrophilic surface modification of microchannel is an effective method to enhance heat transfer and reduce friction pressure drop. Through the data analysis of friction pressure drop and C parameter, it is found that the three most important parameters affecting the two-phase friction pressure drop are mass flux, vapor quality, and surface wettability. Based on flow patterns and pressure drop characteristics obtained from the experiments, a new parameter, named flow order degree, is proposed to account for the overall effects of mass flux, vapor quality, and surface wettability on two-phase frictional pressure drop in microchannels, and a newly developed correlation based on the separated flow model is presented. In the superhydrophilic microchannel, the mean absolute error of the new correlation is 19.8%, which is considerably less than the error of the previous models.

## 1. Introduction

Over the past few years, as technologies have developed, the demand for heat dissipation of small-size and high heat flux devices is growing [1]. Compared with traditional macro-size channels, microchannel heat exchangers have a small size and highly efficient heat transfer [2,3,4,5], and can be used in areas, such as MEMS, space, and high-power concentrated photovoltaic systems. Boiling heat transfer is usually used in the microchannel due to its high heat transfer efficiency [6,7,8,9]. Many experiments have studied the effects of fluid viscosity [10,11,12,13,14,15], microchannel size, heat flux, mass flow rate, and vapor dryness [16,17,18,19,20] on pressure drop, but most of them did not take surface wettability into account. Enoki et al. [21] investigates the experimental study of water boiling heat transfer and pressure drop in a microchannel pipe flowing vertically upwards, observing that the two-phase flow regime predicted in the test section is divided into three modes, namely slug, annular and churning flow regimes, and the analysis of the experimental data reveals that the heat transfer coefficient is significantly higher at higher mass fluxes, but the pressure loss is greater. Kundu et al. [22] studied the flow boiling heat transfer characteristics and pressure drop characteristics of pure refrigerant (R134a), quasi azeotropic mixtures (R410A) and averaged boiling mixtures (R407C) and they found that heat flux and mass velocity significantly affect the heat transfer coefficient and two-phase pressure drop.

The surface modification of microchannel can change the characteristics of heat transfer and flow pressure drop [23,24,25], and the flow patterns on different wettability surface microchannels are very different, which in turn affects the variation of frictional pressure drop inside the microchannels. Our team has previously obtained models that accurately predict common surface microchannels in pressure drop prediction studies but has not explored surface-modified microchannels [26]. In addition, raccooning or hybrid waving of the microchannel is also an effective way to enhance heat transfer [27,28].

Using theoretical models to predict microchannel pressure drop is useful for designing microchannel heat exchangers. For the study of two-phase flow pressure drop, there are two main approaches: the homogeneous flow model and the separated flow model. When the slip velocity between phases is modest, as in bubbly and atomic flows, the first model is expected to be relevant [29]. Most research on how to predict pressure drop in microchannels involves a model based on Lockhart and Martinelli’s model of separated flow (LM model) [30]. In the LM model, Chisholm parameter was presented, which measures the degree to which gas and liquid interact. In the prediction models of different scholars, the Chisholm parameters are different. The parameters, such as vapor quality and mass flux, all affect the interactions between the gas and the liquid in the flow [31,32,33,34,35,36]. Therefore, finding the appropriate correlation of the Chisholm parameter is the key to accurately predicting the pressure drop.

Choi et al. [37] regarded surface wettability as an important factor in pressure loss, because of the surface force. Through visualization of flow patterns, they found that pressure loss in the hydrophilic microchannel was lower than that in the hydrophobic microchannel due to the movement of bubbles and liquid. Zhou et al. [38] conducted an experimental investigation of saturated two-phase flow in the channel using water. From the flow visualization, they found that the dry-out event happened on the common channel at low mass velocity and large heat flux.

Our research team conducted some work on the law and model of flow heat conduction and frictional pressure loss prediction in the common surface microchannel [26] and acquired data on the two-phase pressure drop. The experimental study showed that vapor quality, particularly the poor vapor quality zone, has a major impact on the Chisholm parameter. Then, a new parameter named the superficial gas flux, is introduced to evaluate the overall effects of vapor quality and mass flux on frictional pressure drop, and a new correlation is presented. The effect of surface modification was not considered in that work. However, we know that surface-modified microchannels can significantly improve their heat transfer performance, and their application prospects are widely recognized. So far, there is no good prediction model that can be used to predict the flow pressure drop in microchannels with surface modification. Based on the previous work, this paper will focus on the influence of surface wettability on the pressure drop of microchannel flow to obtain a better prediction model.

## 2. Materials and Methods

### 2.1. Experimental Apparatus and Procedures

**Figure 1:**
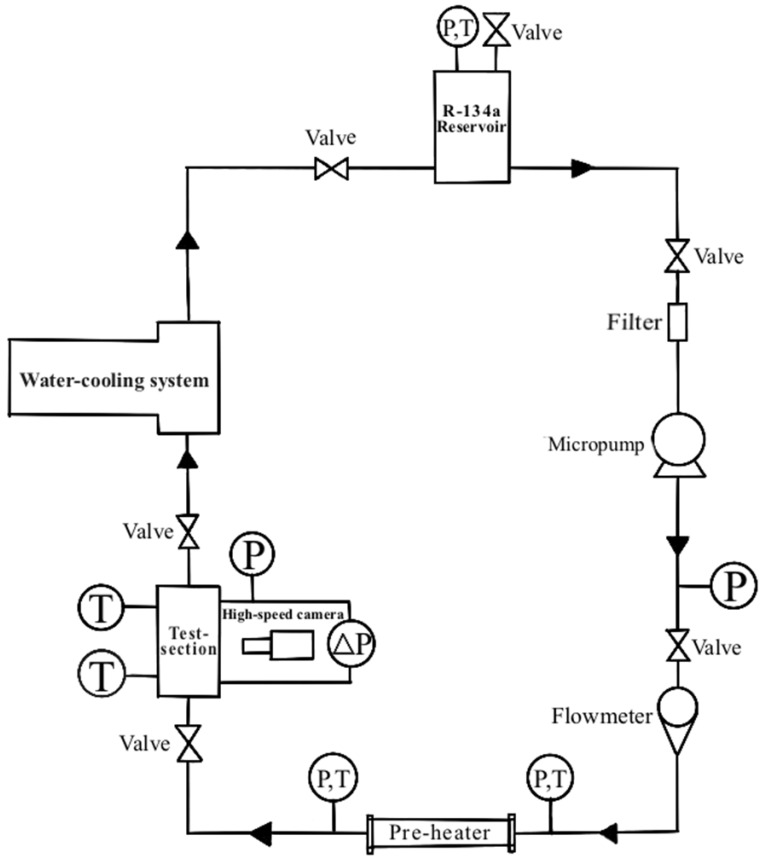
The schematic diagram of the experimental devices.

As shown in Figure 1, the experimental loop consists of a R-134a reservoir, a micropump, a pre-heater, a test section, a high-speed camera and water-cooling system. In the experimental loop, R-134a is pumped into the test section from the reservoir using a micropump after passing through a pre-heater. R-134a is heated as it passes through the pre-heater to adjust the quality of the inlet vapor before flowing into the microchannel test section.

**Figure 2:**
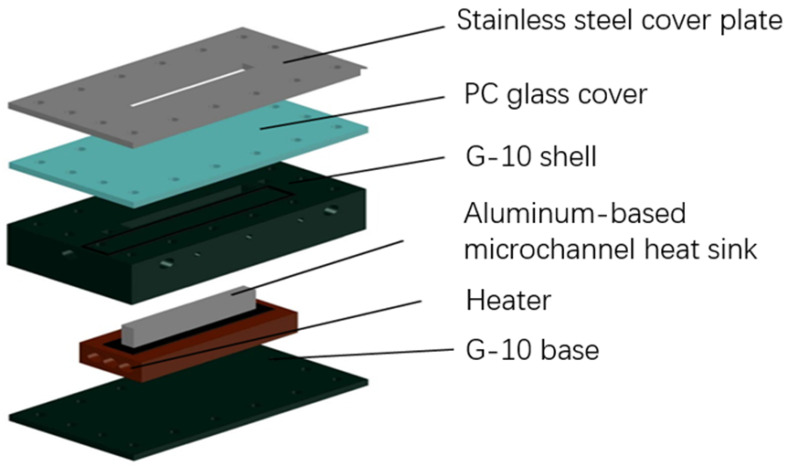
Structure of microchannel test section.

Figure 2 depicts the structure and details of the microchannel test section. There are three microchannels with a width of 1.50 mm, a depth of 0.55 mm, and a total length of 78 mm in the test section.

In the experiment, the aluminum-based microchannel was successfully prepared through electric spark cutting, polishing, chemical corrosion, ultrasonic cleaning, etc. Superhydrophilic surfaces (contact angle of 0°), hydrophilic surfaces (contact angle of 43°) with complex surface hole microstructures, and common surfaces (contact angle of 70°, unmodified) were obtained.

The experiments were conducted under the following test fluid conditions: saturation pressure *Ps* = 5.6 bar, mass flux *G* = 713–1629 kg/m^2^s, heat flux *q* = 7.0–35.1 kW/m^2^, and inlet vapor quality *x_in_* = 0.02–0.17, as seen in Table 1. These experimental conditions were selected in response to our team’s previous research on study and mechanism analysis of the flow boiling and heat transfer characteristics in microchannels with different surface wettability, prediction on pressure drop during flow boiling in common surface microchannels, and the experimental conditions of previous scholars’ pressure drop prediction models.

After the experimental system circuit is set up and the gas tightness is checked, the circuit is evacuated using a vacuum pump, the air conditioning system is switched on and the room temperature is stabilized at around 20 °C. The R-134a refill is then added to the reservoir and the system and after the data have stabilized, the power supply and all devices are started. The light source and the high-speed camera are turned on and the appropriate light intensity, focal length, aperture and other parameters are adjusted. The gear pump is adjusted to the appropriate flow rate and the heating power of the preheating section and the experimental section are also adjusted. We wait again for the data to stabilize and then start the data acquisition instrument, setting the time for each acquisition to two minutes and the data set to 240 groups. During this time, a high-speed camera is used to photograph the flow pattern of the working fluid. After the acquisition and filming is completed, the data are marked and stored on the hard drive, and while waiting for the acquisition to be completed, the data are constantly monitored for any abnormalities. Each time the condition is adjusted, the temperature and pressure drop data of the experimental circuit is stabilized for five minutes before the above steps can be repeated.

A pressure sensor with an error of less than 0.7% measures the pressure of R-134a as it flows in. A differential pressure transmitter is used to measure the test-differential section with an error of less than 1%. Two resistance temperature detectors with an error of less than 0.5 °C are installed at the inlet and exit, and the thermocouples with an error of less than 0.5 °C are placed in the heat sink to monitor surface temperature. For each circumstance, temperatures and pressures are recorded for 2 min at 0.5 s intervals. Data acquisition hardware is used to collect and process the measured data. Table 2 shows the features of the instruments and appliances. The uncertainty of variables is listed in Table 3.

### 2.2. Data Reduction

The frictional pressure drop and the acceleration pressure drop comprise the two-phase pressure drop in the horizontal microchannel. Because the microchannel is horizontal, the gravitational component can be negligible.

(1) $$\Delta p_{tp}=\Delta p_{tp,f}+\Delta p_{tp,a}$$

Equation can be used to determine the component of acceleration (2) [32].

(2) $$\Delta p_{tp,a}=G^{2}\left\{\left[\frac{{\left(1-x_{out}\right)}^{2}}{\rho_{l}\left(1-\alpha_{out}\right)}+\frac{x_{out}^{2}}{\rho_{g}\alpha_{out}}\right]-\left[\frac{{\left(1-x_{in}\right)}^{2}}{\rho_{l}\left(1-\alpha_{in}\right)}+\frac{x_{in}^{2}}{\rho_{g}\alpha_{in}}\right]\right\}$$

where, $x_{out}$ and $x_{in}$ indicate the vapor quality at the inlet and exit, respectively, and $\alpha_{out}$ and $\alpha_{in}$ indicate the void fraction at the inlet and exit, respectively.

Equations (3) and (4) can be used to calculate the vapor quality at the inlet and exit.

(3) $$x_{in}=\frac{h_{l0}+\frac{\dot{P}}{\dot{m}}-h_{l,in}}{h_{tp,in}}$$

(4) $$x_{out}=x_{in}+\frac{\dot{Q}}{\dot{m}h_{tp,out}}$$

From Zivi’s [39] model, the void fraction α can be determined as follows:

(5) $$\alpha =\frac{1}{1+\left(\frac{1-x}{x}\right){\left(\frac{\rho_{g}}{\rho_{l}}\right)}^{\frac{2}{3}}}$$

It is possible to obtain the two-phase frictional pressure drop gradient from the LM model [30].

(6) $${\left(-\frac{d_{P}}{dz}\right)}_{tp,f}={\left(-\frac{d_{P}}{dz}\right)}_{l}+C{\left[{\left(-\frac{d_{P}}{dz}\right)}_{l}{\left(-\frac{d_{P}}{dz}\right)}_{g}\right]}^{\frac{1}{2}}+{\left(-\frac{d_{P}}{dz}\right)}_{g}$$

where, *C* is the Chisholm parameter measuring the degree of gas/liquid interaction.

In the LM model [30], the two-phase multiplier is defined:

(7) $$\phi_{l}^{2}=\frac{{\left(\frac{d_{P}}{dz}\right)}_{tp,f}}{{\left(\frac{d_{P}}{dz}\right)}_{l}}$$

The liquid frictional pressure drop ratio and the gas frictional pressure drop ratio can be given from:

(8) $${\left(\frac{d_{P}}{dz}\right)}_{l}=\frac{2f_{l}{\left(1-x\right)}^{2}G^{2}}{D_{h}\rho_{l}}$$

(9) $${\left(\frac{d_{P}}{dz}\right)}_{g}=\frac{2f_{g}x^{2}G^{2}}{D_{h}\rho_{g}}$$

where, $f_{l}$ indicates the friction factor for the liquid phase, and $f_{g}$ indicates the friction factor for the gas phase.

The local heat transfer coefficients are calculated as follows:

(10) $$H_{z}=\frac{q}{T_{w,z}-T_{sat,z}}$$

$T_{w,z}$ is the local wall temperature, and $T_{sat,z}$ is the saturation temperature based on local saturation pressure.

The average heat transfer coefficient is obtained by integrating the local heat transfer coefficients at each location as follows:

(11) $$H_{ave}=\frac{1}{L}\int_{0}^{L}H\left(z\right)dz$$

## 3. Experimental Results

### 3.1. The Effect of Surface Wettability on Heat Transfer Performance

Before formally investigating the pressure drop characteristics and flow patterns of microchannels with different wetting surfaces, this paper first investigates the heat transfer characteristics of microchannels with different wettability surfaces in order to confirm whether the surface modification of microchannels has an enhanced effect on the heat transfer of microchannels. This is to confirm whether surface modification of microchannels is useful for microchannel design applications and whether it is necessary to study the pressure drop characteristics of microchannels with different wettability surfaces.

**Figure 3:**
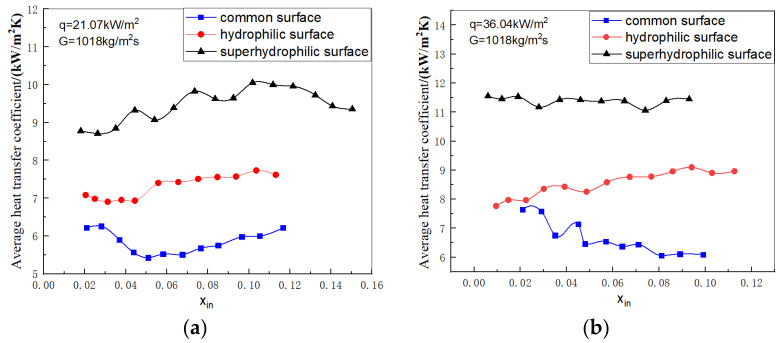
Variation of the average heat transfer coefficient versus inlet vapor quality in three different wettability surface microchannels. (**a**) q = 21.07 kW/m^2^, *G* = 1018 kg/(m^2^s). (**b**) q = 36.06 kW/m^2^, *G* = 1018 kg/(m^2^s).

Figure 3a shows the curve of the average heat transfer coefficient with inlet vapor quality for three different wettability surfaces at a heat flow density of 21.07 kW/m^2^ and a mass flow rate of 1018 kg/(m^2^s). At low inlet vapor quality, the flow pattern is mainly bubbly in this interval and heat transfer is dominated by nucleation boiling.

In Figure 3a, the curve for the normal surface microchannel is the lowest because the normal surface does not have the same surface microstructure cavities as the hydrophilic and superhydrophilic surfaces and has fewer vaporization cores in comparison. Additionally, because the poor wettability of the normal surface makes it difficult to detach the small bubbles generated on the wall surface by moving close to the wall surface, the contact area between the wall surface and the liquid is reduced. The superhydrophilic surface has better wettability and is closer to the liquid than the hydrophilic surface, making it easier for the bubbles to detach and the heat transfer coefficient greater, so the curve for the superhydrophilic surface microchannel in Figure 3a is higher than the curve for hydrophilic. The average heat transfer coefficient of the superhydrophilic microchannel is about 64% higher than the average heat transfer coefficient of the normal surface microchannel at best, and the hydrophilic surface is about 26.7% higher than the normal surface at best. In Figure 3b, it can be seen that at high heat flow densities, the value of the average heat transfer coefficient of the superhydrophilic surface is approximately 80% higher than the normal surface at best.

Through this investigation we have found that hydrophilic surface modification of microchannels can improve the average heat transfer coefficient and heat transfer performance of microchannels, with the highest heat transfer coefficient in superhydrophilic microchannels. Thus, we focus on superhydrophilic microchannels in our study of pressure drop characteristics and flow pattern. Hydrophilic surface modification of microchannels is an effective method of enhancing heat transfer and this paper concentrates on the effect of wettability on pressure drop under conditions where heat transfer performance is improved, thus defining the range of conditions for this study.

### 3.2. Flow Pattern

It is known that flow pattern reflects the characteristics of flow, which is closely related to heat transfer and pressure drop. However, there is no work that can predict the frictional pressure drop through the microscopic morphology of the flow pattern. In this paper, a fast-moving camera is utilized to take many flow pattern diagrams under different working conditions in superhydrophilic and common microchannels.

**Figure 4:**
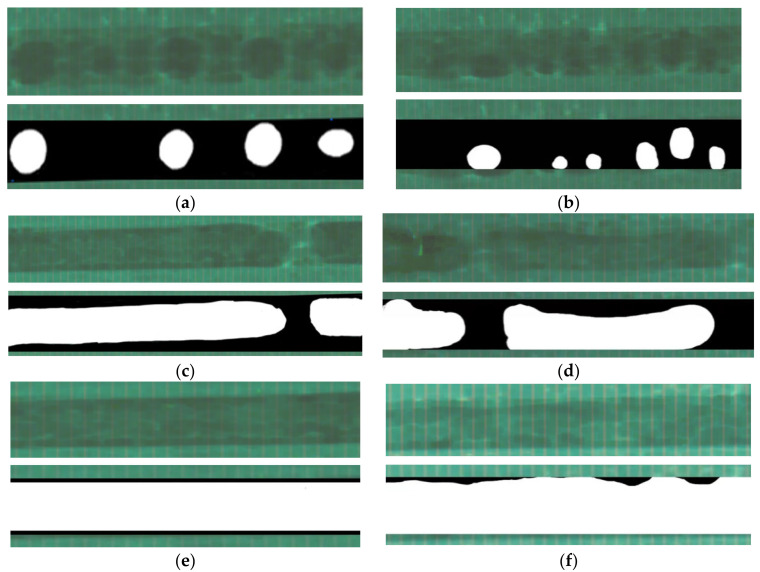
Typical two-phase flow pattern in superhydrophilic and common microchannels. (**a**) Bubbly flow in superhydrophilic microchannel, *q* = 7.023 kW/m^2^, *G* = 1629 kg/(m^2^s), *x_in_* = 0.023. (**b**) Bubbly flow in common microchannel, *q* = 7.023 kW/m^2^, *G* = 1629 kg/(m^2^s), *x_in_* = 0.023. (**c**) Slug flow in superhydrophilic microchannel, *q* = 21.074 kW/m^2^, *G* = 1018 kg/(m^2^s), *x_in_* = 0.064. (**d**) Slug flow in common microchannel, *q* = 21.074 kW/m^2^, *G* = 1018 kg/(m^2^s), *x_in_* = 0.064. (**e**) Ring flow in superhydrophilic microchannel, *q* = 21.074 kW/m^2^, *G* = 1018 kg/(m^2^s), *x_in_* = 0.125. (**f**) Ring flow in common microchannel, *q* = 21.074 kW/m^2^, *G* = 1018 kg/(m^2^s), *x_in_* = 0.125.

First, the visual experimental study found that bubbly flow, slug flow, and ring flow patterns appeared in the superhydrophilic microchannel produced, as shown in Figure 4. Bubbly flow occurred at low heat fluxes and low vapor quality. Bubble volume increased as vapor quality and heat flux rose, and slug flow occurred. The long bubbles collapsed together at a particularly higher degree of vapor quality and heat fluxes, and ring flow occurred [40,41,42]. These results are same as that shown in Figure 4.

Moreover, under the same working conditions, flow patterns in superhydrophilic microchannel are different from that in the common microchannel we observed previously. The difference is the order degree of bubbles.

For the bubbly flow, bubbles in superhydrophilic microchannel are easier to separate, so most of the bubbles in superhydrophilic microchannel are distributed in the center of the microchannel and do not contact the surface (as shown in Figure 4a); it is relatively regular and orderly. However, the distributions of bubbles and liquid are more random in the common microchannel. The order degree of bubbles in the common microchannel is low (as shown in Figure 4b), because the poor wettability of the common microchannel makes it difficult for bubbles to separate from the wall, and the friction between the gas–liquid interaction is large [43].

For the slug flow, it is found both in superhydrophilic microchannel and common microchannel under the conditions of *q* = 21.074 kW/m^2^, *G* = 1018 kg/(m^2^s), and *x_in_* = 0.064 (as shown in Figure 4c,d). In superhydrophilic microchannel, the bubbles are distributed in the center of the microchannel. However, in the common surface microchannel the shape and position of bubbles are irregular, and the order degree of bubbles is low, which leads to uneven bubble film thickness.

Similarly, for the ring flow, it is found both in the superhydrophilic microchannel and common microchannel under the conditions of *q* = 21.074 kW/m^2^, *G* = 1018 kg/(m^2^s), and *x_in_* = 0.125 (as shown in Figure 4e,f). In the superhydrophilic microchannel, the liquid film is smooth and stable without drying out. The thickness of the liquid film attached to the microchannel surface has not changed significantly. The bubbles are almost stable in the center of the microchannel. The gas–liquid two-phase distribution is relatively orderly. However, in the common microchannel, bubbles randomly appeared on the wall and dried out of the wall by moving, which is a drying phenomenon [44]. Gas and liquid are randomly distributed. The liquid film is unstable and disordered, and the order degree of bubbles is low.

The above discussion reveals that the three flow patterns captured in the conditions studied here are bubbly flow, slug flow, and ring flow. The two-phase flow in a well-wetted microchannel is more ordered, with bubbles always distributed in the center of the microchannel and the liquid film always distributed near the surface of the microchannel and of uniform thickness. In poorly wetted microchannels, the two-phase flow is more random, with bubbles appearing randomly near the microchannel walls causing dryness and the film distribution is also more random and uneven. The surface wettability of the microchannels affects the orderly or random distribution of the gas–liquid two-phase flow pattern.

### 3.3. Frictional Pressure Drop

The most significant component of the two-phase flow pressure loss is the frictional pressure drop which is heavily correlated with vapor quality.

**Figure 5:**
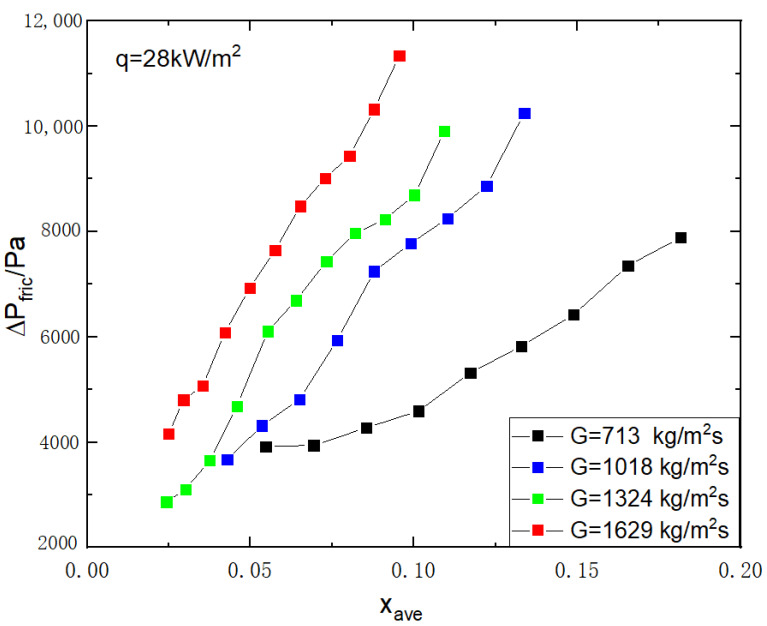
Variation of two-phase frictional pressure drop versus average vapor quality at different mass flux in the superhydrophilic microchannel (contact angle $\theta =0{}^{\circ},q=28\;kW/m^{2}$).

The frictional pressure drop in the superhydrophilic microchannel varies depending on the average vapor quality, as shown in Figure 5. The frictional pressure drop increases as vapor quality increases and is influenced by mass flux with a positive correlation. The trend of frictional pressure drop in the superhydrophilic microchannel is the same as that in the common microchannel from our previous research, Huang et al.’s study [26].

**Figure 6:**
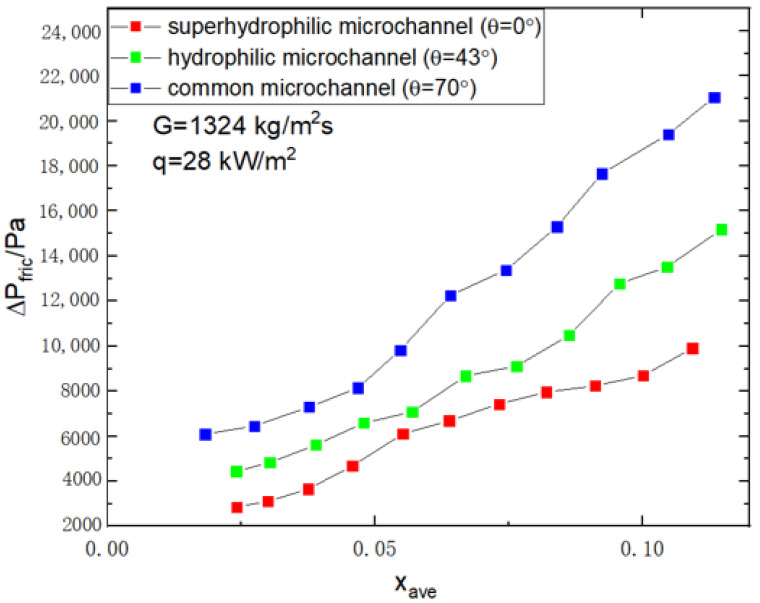
Variation of two-phase frictional pressure drop versus average vapor quality in the microchannels with different surface wettability ($G=1324\;kg/m^{2}s,q=28\;kW/m^{2}$).

Figure 6 reveals the effect of different wettability of the surface microchannels on two-phase frictional pressure drop; the two-phase frictional pressure drop is smaller for well-wettable microchannels. The effect of surface wettability on two-phase frictional pressure drop is more significant in conditions with high vapor quality, because the flow pattern in the microchannel is ring flow in conditions with high vapor quality, and the hydrophilic modification makes it easier for the bubbles in the microchannel to detach from the wall and stabilize in the center of the microchannel while reducing the occurrence of dry-out. Hence, the reduction in the two-phase frictional pressure drop is more obvious in the superhydrophilic microchannel.

In the different wettability microchannels, mass flux and vapor quality both have a positive effect on two-phase frictional pressure drop. The two-phase frictional pressure drop is smaller in well-wetted microchannels and the hydrophilic modification of the microchannels improves the two-phase frictional pressure drop more significantly in conditions of high vapor quality

### 3.4. Vapor Quality’s Impact on the Chisholm Parameter

**Figure 7:**
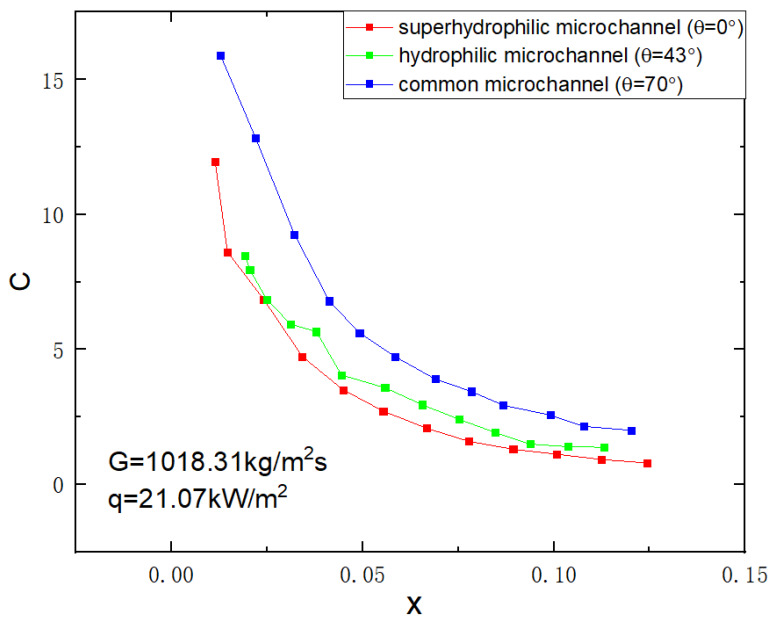
Variation of C in different surface wettability microchannels versus vapor quality ($G=1018.31\;kg/m^{2}s$, $q=21.07\;kW/m^{2}$).

The Chisholm parameter C (as shown in Formula (6)) determines the frictional pressure drop between gas–liquid phases. It is found that the vapor quality is inversely proportional to C, as shown in Figure 7.

When *x* < 0.7, *C* drops rapidly as vapor quality increases, and when *x* > 0.7, *C* tends to be constant. Combined with the visual flow pattern (as shown in Figure 4), as *x* increased, we found the bubbles became more, bubbly flow became slug flow, and ring flow with the bubbles slowly fused. Because the bubbles in the superhydrophilic microchannel were almost in the center of the microchannel and the liquid was at the wall, the two phases of gas and liquid became more ordered.

In general, the value of the Chisholm parameter is smaller for well-wetted microchannels, which is consistent with the conclusion that the friction between the gas–liquid interaction is smaller due to the higher flow order degree of well-wetted microchannels as analyzed in the flow pattern earlier in this study. Through this subsection, we have found that C is inversely related to vapor quality and also correlates with wettability, and these correlations are found to be of great help in the formulation of new parameters and the improvement of pressure drop models later on.

## 4. New Correlation

### 4.1. Evaluation of Correlations

In order to establish the two-phase flow pressure drop correlation in the surface-modified microchannels, the main two-phase flow models are evaluated using our experimental data.

**Figure 8:**
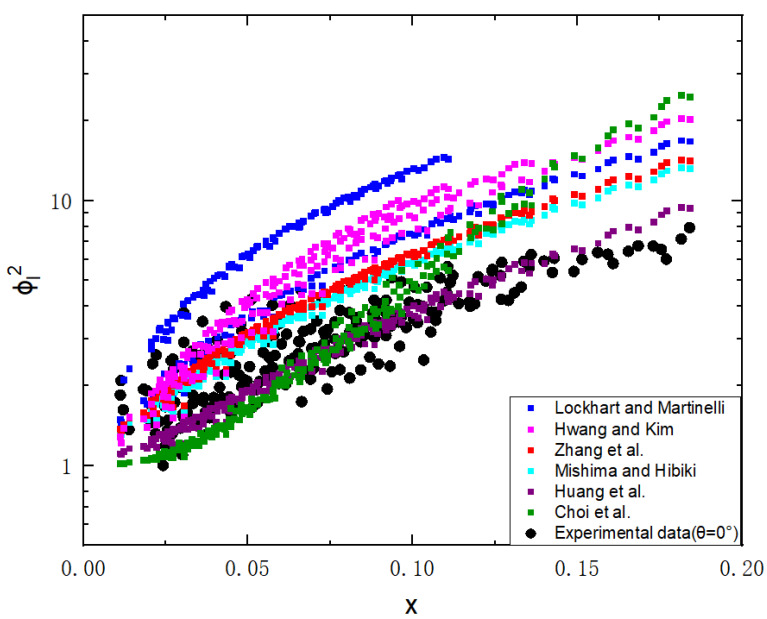
Measured two-phase multiplier and previous models’ two-phase multiplier in superhydrophilic microchannels [26,30,32,34,36,45].

Two-phase frictional multiplier versus the vapor quality is shown in Figure 8. The different models which modified the LM model are used to calculate the predicted values. Table 4 lists details of the models and their accuracy under our experimental conditions. The mean absolute error (MAE), which is defined as the measure of the precision of correlations, is,

(12) $$MAE=\frac{1}{N}\sum \frac{\left|\Delta P_{pred}-\Delta P_{exp}\right|}{\Delta P_{exp}}\times 100\%$$

**Figure 9:**
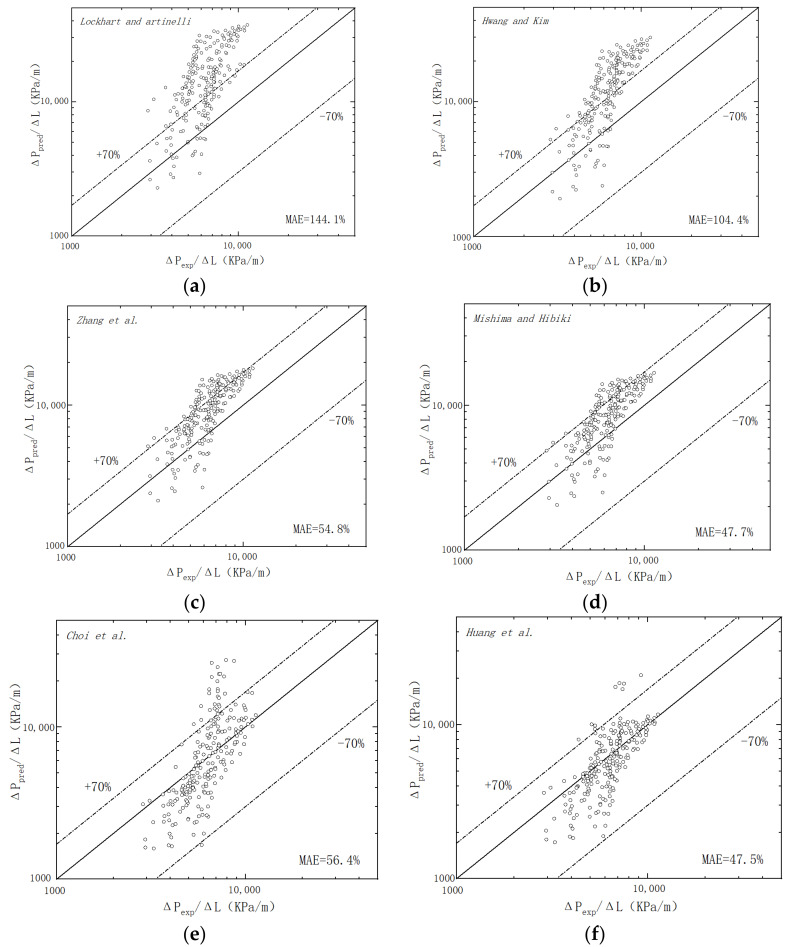
Comparison between the experimental frictional pressure drop values in superhydrophilic microchannels and previous models (**a**) Lockhart and Martinelli [30], (**b**) Hwang and Kim [45], (**c**) Zhang et al. [34], (**d**) Mishima and Hibiki [32], (**e**) Choi et al. [36] and (**f**) Huang et al. [26] (our previous research).

Figure 9 shows the measured pressure drop values compared to the predictions based on the six correlations mentioned above. Figure 8 and Figure 9 both reveal that most of the models overpredict the two-phase friction multiplier compared to the experimental values.

The LM model [30], widely used for big-size channels, is the most inconsistent with the experimental results. Due to the effect of size and surface in microchannels, it no longer applies to small-size channels.

The models of Mishima and Hibiki [32], Lockhart and Martinelli [24], Hwang and Kim [45], and Zhang et al. [34] overestimated the frictional multiplier. The models of Mishima and Hibiki [32], Zhang et al. [34], and Huang et al. [26] share similar modalities. Huang et al. [26] introduced a new parameter, named the superficial gas flux, to evaluate the overall effects of vapor quality and mass flux on two-phase frictional pressure drop in microchannels, with a mean absolute error of 47.5%. Among the existing correlations, Huang et al.’s correlation [26] predicted the experimental data most accurately. The model of Mishima and Hibiki [32], with a mean absolute error of 47.7%, applies only to mini-channel bubbly flow, while there are other flow patterns, such as slug flow and ring flow, in this experiment.

Hwang and Kim’s correlation [45] contains the influence of channel diameter, surface tension, and Reynolds number on the two-phase flow pressure loss in the channels. However, the model of Hwang and Kim [45] inaccurately predicted the experimental value, with a mean absolute error of 104.4%. They experimented in a 0.244 mm circular stainless-steel tube with a mass flux between 480 and 950 kg/m^2^s. The shape, size, and wettability of their experimental microchannel differed too much from the experimental channel of this experiment.

Zhang et al.’s correlation [34], with a mean absolute error of 54.8%, covered several experimental conditions. They collected the experimental results of numerous researchers with different hydraulic diameters with wide ranges and numerous varieties of working fluids, except considering the effect of surface wettability.

The model of Choi et al. [36] gives a relatively accurate prediction with a mean absolute error of 56.4%. Their working fluid is FC-72, which has similar properties to R-134a. Reynolds and Weber numbers were used in the correlation. The model includes the effects of viscous and inertial forces but does not include the impact of surface wettability.

Compared to the evaluation of six correlations, the prediction of the model of Huang et al. [26] is most similar to the experimental value. To further investigate the effect of surface wettability on the model, this work will develop the model based on the experimental data, flow pattern diagram, and order analysis results presented previously.

### 4.2. Newly Developed Correlation

A new correlation is developed to improve the accuracy of two-phase flow pressure drop prediction in microchannels. Owing to the experimental results shown above, mass flux, vapor quality, and the surface wettability of microchannels are the three important parameters that affect the two-phase flow pressure drop.

In Section 3.3 and Section 3.4, we found that the Chisholm parameter is inversely proportional to vapor quality x and positively correlated with mass flux G in microchannels.

In Section 3.2, we found that surface wettability affects the degree of gas–liquid order in the flow, and thus affects the friction pressure drop of gas–liquid two phases. Next, we want to establish the connection between surface wettability and the Chisholm parameter through the contact angle. The contact angle θ is an important physical quantity for measuring surface wettability and surface tension. The relationship between the solid surface energy parameter $\gamma_{s}$, and the liquid surface energy parameter $\gamma_{l}$ is [46]:

(13) $$\gamma_{l}=2\gamma_{s}*\frac{1}{\sqrt{1+\sin^{2}\theta }+\cos\theta }$$

The surface energy and surface tension parameters describe the surface structure characteristics and surface wettability from the energy and mechanical perspectives, respectively.

Therefore, in this paper, we define an impact factor $W_{\theta }$, which describes the effect of contact angle θ on two-phase frictional pressure drop:

(14) $$W_{\theta }=\frac{1}{\sqrt{1+\sin^{2}\theta }+\cos\theta }$$

Then, a new parameter, the flow order degree $D_{ord}$ is presented to account for the combined effects of mass flux, surface wettability and vapor quality in two-phase microchannel frictional pressure drop.

(15) $$D_{ord}=\frac{G}{x*\rho_{l}}*\frac{1}{\sqrt{1+\sin^{2}\theta }+\cos\theta }$$

By evaluating the pressure drop prediction models of six previous scholars, we found that Zhang et al. [28] collected a large amount of experimental data and proposed a new correlation equation using the artificial neural network, and Huang et al.’s correlation [26] considered the combined effect of vapor quality and mass flux on the two-phase frictional pressure drop in microchannels. In this paper, the Chisholm parameters in Zhang et al. ’s correlation [34] and Huang et al.’s correlation [26] are improved, and $C_{modify}$ is defined as a function of $D_{ord}$:

(16) $$C_{modify}=2.1\left[1-\exp\left(-\frac{0.358}{La}\right)\right]\left(C_{1}*D_{ord}+C_{2}\right)$$

In the 296 experimental data points under mass flux of 713–1629 kg/m^2^s, heat flux of 0–35.12 kW/m^2^ and vapor quality of 0–0.2, the constants of $C_{1}$ and $C_{2}$ were calculated using the least error square method.

(17) $$C_{1}=0.34,C_{2}=0.15$$

### 4.3. Evaluation of New Correlation

**Figure 10:**
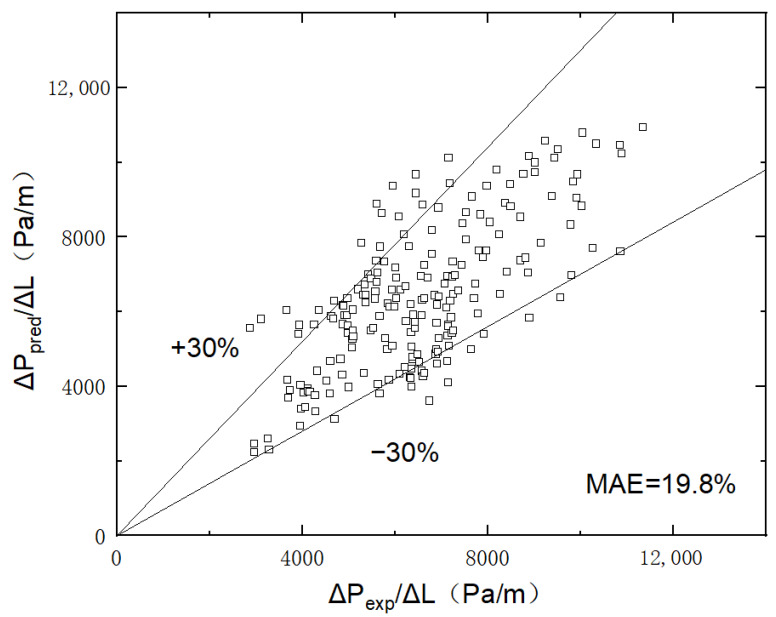
Comparison of frictional pressure drop value with predictions of the new correlation in superhydrophilic microchannel (the contact angle of 0°).

As shown in Figure 10, the newly developed correlation with a mean absolute error of 19.8% is well suited to the experimental data.

**Figure 11:**
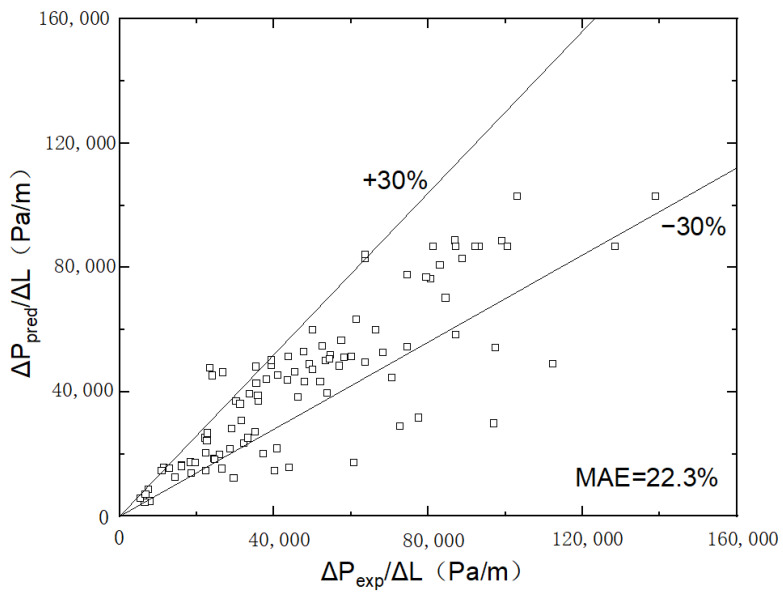
Comparison of frictional pressure drop value with predictions of the new correlation in hydrophilic microchannel (the contact angle of 43°).

To verify the precision of the new correlation in predicting microchannels on different wettability surfaces, hydrophilic microchannel data with a contact angle of 43° were examined with a mean absolute error of 22.3%. Figure 11 shows that the precision of the model is enhanced. Most data points fall within a 30% range of variance. The newly developed correlation is highly efficient.

## 5. Conclusions

Experiments primarily investigate the two-phase flow pattern and pressure drop prediction in different surface wettability microchannels using R-134a. The results are summarized as follows:

1. Hydrophilic surface modification of microchannels can improve the average heat transfer coefficient and heat transfer performance of microchannels, with the highest heat transfer coefficient in superhydrophilic microchannels. Hydrophilic surface modification of microchannels can also reduce two-phase frictional pressure drop. The frictional pressure drop increases with increasing vapor quality and mass flux in microchannels with different surface wettability.
2. The surface wettability of the microchannels affects the orderly or random distribution of the gas–liquid two-phase flow pattern. The two-phase flow in a well-wetted microchannel is more ordered, with bubbles always distributed in the center of the microchannel and the liquid film always distributed near the surface of the microchannel and of uniform thickness. In poorly wetted microchannels, the two-phase flow is more random, with bubbles appearing randomly near the microchannel walls causing dryness and the film distribution is also more random and uneven.
3. This paper evaluated the pressure drop prediction models of six previous scholars and found that most of them overestimated the frictional pressure drop and did not consider the surface wettability. Compared to the evaluation of six correlations, the prediction of the models of Huang et al. [26] and Zhang et al. [34] are more accurate to the experimental value, and they consider a wide range of working conditions. Thus, this paper improves the model based on their prediction models.
4. A new parameter, named flow order degree $D_{ord}$, is presented to account for the overall effects of mass flux, vapor quality, and surface wettability on two-phase frictional pressure drop in microchannels, and a newly modified Chisholm parameter is presented. The newly developed correlation with $C_{modify}$ predicts the two-phase flow pressure drop in the superhydrophilic microchannel quite well with a mean absolute error of 19.8%.

## Figures and Tables

**Table 1:** Experimental conditions.

Parameter	Range
Mass flux *G*	713–1629 kg/m^2^s
Heat flux *q*	7.0–35.1 kW/m^2^
Inlet vapor quality *x_in_*	0.02–0.17

**Table 2:** Instruments and appliances.

Quantity	Apparatus	Range	Accuracy
Temperature	T-type thermocouple	−50–250 °C	±0.5 °C
Pressure	Piezoelectric transducers	0–20 bar	±0.7%
Pressure drop	Differential pressure transmitter	0–10 bar	±1.0%
Mass flux	Flowmeter	0–2200 kg/m^2^s	±0.5%

**Table 3:** Uncertainty of variables.

Parameter	Uncertainty
Pressure	0.7%
Pressure drop	1.0%
Fluid temperature	0.5 °C
Wall temperature	0.5 °C
Heat flux	0.5%
Mass flux	0.5%
Vapor quality	7.2%
Contact angle	1.0%

**Table 4:** Existing correlations and MAE.

Reference	The Chisholm Parameter	MAE
Lockhart and Martinelli [30]	$C_{vv}=5$ $C_{tv}=10$ $C_{vt}=12$ $C_{tt}=20$	144.1%
Hwang and Kim [45]	$C=0.227{Re}_{lo}^{0.452}X^{-0.32}N_{conf}^{-0.82}$	104.4%
Zhang et al. [34]	$C=21[1-\exp\left(-\frac{0.358}{La}\right)]$	54.8%
Mishima and Hibiki [32]	$C=21[1-\exp\left(-0.319D_{h}\right)]$	47.7%
Choi et al. [36]	$C=0.05{Re}_{lo}^{0.68}X^{-1.32}{We}_{lo}^{-0.34}$	56.4%
Huang et al. [26] (our previous research)	$C=21[1-\exp\left(-\frac{0.358}{La}\right)](0.06548j_{g}+0.17033)$	47.5%

## Data Availability

The data presented in this study are available on request from the corresponding author. The data are not publicly available due to privacy.

## Nomenclature

G	mass flux [kg/m^2^s]
q	heat flux [kW/m^2^]
x	thermodynamic equilibrium vapor quality [-]
p	pressure [Pa]
ρ	density [kg/m^3^]
α	void fraction [-]
$\dot{P}$	heating power of the preheating section [W]
$\dot{m}$	mass flow rate [kg/s]
h	enthalpy [J/kg]
Re	the Reynolds number [-]
We	the Weber number [-]
v	flow velocity [m/s]
$D_{ord}$	the flow order degree [m/s] $D_{ord}=\frac{G}{x*\rho_{l}}*\frac{1}{\sqrt{1+\sin^{2}\theta }+\cos\theta }$
H	heat transfer coefficient [W/m^2^K]
$\dot{Q}$	heating power of the test section [W]
z	coordinate along microchannel [mm]
*C*	the Chisholm parameter [-]
σ	surface tension of the liquid [N/m]
$\phi_{l}^{2}$	the two-phase multiplier
f	friction factor [-]
$D_{h}$	hydraulic diameter [mm]
La	the Laplace number [-] $La=\sqrt{\frac{\sigma }{g\left(\rho_{l}-\rho_{g}\right)D_{h}^{2}}}$
γ	the surface energy parameter [N/m]
θ	contact angle [°]
Δ	difference [-]
MAE	mean absolute error [-]
T	temperature [K]
X	the Martinelli parameter [-] $X^{2}=\frac{{\left(\frac{dP}{dz}\right)}_{l}}{{\left(\frac{dP}{dz}\right)}_{g}}$
L	channel length [mm]
$j_{g}$	superficial gas flux [m/s] $j_{g}=\frac{Gx}{\rho_{g}}$
**Subscripts**	
a	accelerational
ave	average
exp	experimental
fric	frictional
g	saturated vapor
l	saturated liquid
s	solid
tv	turbulent liquid-laminar vapor
vv	laminar liquid-laminar vapor
z	coordinate along microchannel
a	accelerational
in	microchannel inlet
out	microchannel outlet
pre	predicted
tot	total
tp	two-phase
lo	liquid only
tt	turbulent liquid-turbulent vapor
vt	laminar liquid-turbulent vapor
w	wall
